# AdipoRon and Other Adiponectin Receptor Agonists as Potential Candidates in Cancer Treatments

**DOI:** 10.3390/ijms22115569

**Published:** 2021-05-25

**Authors:** Ersilia Nigro, Aurora Daniele, Alessia Salzillo, Angela Ragone, Silvio Naviglio, Luigi Sapio

**Affiliations:** 1 Dipartimento di Scienze e Tecnologie Ambientali Biologiche Farmaceutiche, Università degli Studi della Campania “Luigi Vanvitelli”, 81100 Caserta, Italy; ersilia.nigro@unicampania.it (E.N.); aurora.daniele@unicampania.it (A.D.); 2 CEINGE-Biotecnologie Avanzate Scarl, 80145 Napoli, Italy; 3 Dipartimento di Medicina di Precisione, Università degli Studi della Campania “Luigi Vanvitelli”, 80138 Napoli, Italy; alessia.salzillo@unicampania.it (A.S.); angela.ragone@unicampania.it (A.R.); luigi.sapio@unicampania.it (L.S.)

**Keywords:** adiponectin, AdipoRon, neoplasms, therapy, osteosarcoma

## Abstract

The high mortality rate together with an ever-growing number of annual cases have defined neoplastic disorders as “the real 21st-century disease”. Its dubious distinction also results from conventional therapy failure, which has made cancer an orphan disease. Therefore, innovative and alternative therapeutic strategies are mandatory. The ability to leverage human naturally occurring anti-tumor defenses has always represented a fascinating perspective, and the immuno blockage approval in cancer treatment represents in timeline the latest success. As a multifunctional organ, adipose tissue releases a large amount of adipokines having both carcinogenic and antitumor properties. The negative correlation between serum levels and risk for developing malignancies, as well as the huge number of existing preclinical studies, have identified adiponectin as a potential anticancer adipokine. Nevertheless, its usage in clinical has constantly clashed with the inability to reproduce a mimic synthetic compound. Between 2011 and 2013, two distinct adiponectin receptor agonists were recognized, opening new scenarios even in cancer. Here, we review the first orally active adiponectin receptor agonists AdipoRon, from the discovery to the anticancer evidence. Including our latest findings in osteosarcoma models, we summarize AdipoRon and other existing agonists state-of-art, questioning about the feasibility assessment of this strategy in cancer treatment.

## 1. Introduction

Adipose tissue (AT) is currently considered a multifunctional organ involved in different cellular processes [1]. Initially considered as energy reservoir providing fuel for peripheral organs, adipose tissue has been recognized as a comprehensive functional endocrine organ since the discovery of leptin in 1994 [2]. Through the adipokines production; indeed, AT is actively implicated in countless metabolic and non-metabolic functions, which include, besides the energy storage, thermoregulation and immune responses [3].

With approximately 0.01% of total protein, adiponectin (Acrp30) constitutes one of the most abundant serum-related adipokines [4]. Aside from the direct involvement in glucose and fatty acids homeostasis, several studies have highlighted the relevance of this specific adipokine in obesity-associated cardiovascular diseases [5]. Nevertheless, heart failure does not represent the unique pathological condition in which Acrp30 could exert beneficial effects. Dysregulations in Acrp30 serum levels, for instance, is often recognized in the event of metabolic disorders, immunodeficiencies and malignancies [6,7,8]. Moreover, hypoadiponectinemia has inversely been associated with prognosis even in cancer [9].

The high degree in relapsing, together with its propensity to easily develop resistance, make neoplastic diseases the second leading cause of death worldwide [10]. Regrettably, reverting this trend does not appear to be expected in the near future. Therefore, providing further options in cancer treatment are continuously demanded to increase both progression-free and overall survival.

Recently, a clear interplay between adipose tissue and cancer development and/or progression has been highlighted [11]. In this regard, if from one side adipose tissue dysfunction is closely relevant to increasing morbidity and mortality of cancer patients, on the other side, endocrine disorder of AT associated with an abnormal adipokine secretion has been observed in various cancer types [12]. Growing evidence has in fact proved how Acrp30 can have pleiotropic effects in different cancer models, affecting both proliferation and death [13,14,15]. Although conflicting findings are not lacking, the most notable Acrp30 feature in tumorigenesis is to play a protective role, as extensively reported in breast, colon, prostate, liver and endometrial cancers [16,17].

In view of the foregoing, Acrp30-based therapy has been proposed as a potential pharmacological approach in both cancer prevention and management [9,18]. Nevertheless, intrinsic Acrp30 limitations, such as the heavy molecular mass and the reduced half-life/stability, have always hampered its usage in cancer, and more generally in clinical. Identification of the highly conserved complement factor C1q-like globular domain (gAd), as a plausible Acrp30 active and binding site, has completely changed methodological design of the Acrp30-derived compounds over the years, making currently available different druggable options [19]. Among the others, adiponectin receptor agonists surely represent the most promising Acrp30-related compounds. Nevertheless, whilst some adiponectin receptor agonists’ properties have been well-documented by several studies and even recent reviews, such as anti-obesity, anti-diabetic, anti-depressant, anti-ischemic and anti-hypertrophic features, initial findings propose this class of molecules as potential anticancer agents [20]. Considering that no review currently affords a comprehensive overview about this potentially novel pharmacological application, we designed this essay with the aim of providing an easy state-of-art and a quick look about the strengths and weaknesses of this intriguing topic. Specifically, our emphasis will mainly be dedicated to AdipoRon without, however, neglecting the other agonists. Collecting the latest tumor-related findings, we will evaluate both design and experimental status of Acrp30 agonists, outlining the molecular mechanisms by which AdipoRon exerts antitumor consequences in cancer models. As a testing ground of the devised experimental models, we will also report our recent published findings in which AdipoRon affects growth and, to a lesser extent, death in human osteosarcoma cells. Finally, based on the most consistent results, we will try to define how far ahead is the employment of this therapeutic strategy in cancer treatment.

## 2. A Brief Report on the Adiponectin-Related Properties

Physiologically produced by adipose tissue, Acrp30 is secreted in huge quantities enough to represent about 0.01% of all the serum proteins. Acrp30 is encoded by adipose most abundant gene transcript1 (*ApM1*) localized on the long arm of chromosome 3 in position 27 (locus 3q27), composed by 3 exons and 2 introns. The derived protein is a 244 amino acid cytokine comprised of an N-terminal signal sequence, a variable region, a collagenous domain, and a C-terminal globular domain [21]. Acrp30 is synthesized as a monomer by the adipose cells which assemble to form multimers of diverse molecular weight. The three main isoforms of Acrp30 are:

▪ low molecular weight (LMW), composed by three monomers;
▪ middle molecular weight (MMW), composed by the association of two trimers;
▪ high molecular weight (HMW), composed by eight or more monomers and with a molecular weight > 250 kDa, it represents the most biologically active monomers.

Acrp30 carries out its pleiotropic functions through two widely expressed receptors, named AdipoR1 and AdipoR2, which are structurally similar in both sequence (66.7% of homology) and binding site (86%) [22,23,24]. Originally classified as G-protein-couplet receptors (GPCRs), the recent AdipoR1 and AdipoR2 crystallography has instead revealed significant conformational discrepancies, defining a novel class of receptor structure [25]. Primarily, the seven-transmembrane helices build in this case a central cavity in which three distinct histidine residues bond a zinc ion forming a structure that could be essential in both adiponectin-stimulated AMPK phosphorylation and UCP2 upregulation. Moreover, despite the fact that neither co-crystallization nor binding sites localization and/or characterization have been performed yet, the extracellular layer remains the most likely candidate for ligand–receptor interaction [22].

More recently, T-cadherin, also referred to as Cadherin-13, has also been identified as third putative Acp30 receptor [26]. As a calcium-dependent adhesion molecule, T-cadherin contains an unusual glycosylphosphatidylinositol (GPI) domain, which serves as a membrane-anchoring site. Given this particular structure, T-cadherin can recognize both medium and high Acrp30 oligomers acting as deposit or co-receptor [27].

Upon binding to its receptors, Acrp30 induces the recruitment of the adaptor protein APPL1, thereby activating a plethora of downstream signaling pathways controlling cell survival, cell growth and apoptosis. From a molecular standpoint, Acrp30-receptors binding provokes multiple pathways activation, which primarily includes AMP-activated protein kinase (AMPK) and peroxisome proliferator-activated receptor α (PPARα) [28]. The activation of AMPK, a central sensor and regulator of cellular energy, in turn, stimulates the expression of p21 and p53, and phosphorylates p53 to initiate cell cycle arrest, senescence and apoptosis. Additional studies demonstrated the inhibitory effects of Acrp30 on the PI3K/AKT/mTOR pathway, which leads to a cascade of events resulting in a blockade of cell survival, growth and proliferation. Acrp30 signaling also activates the MAPK cascade, which involves cJNK, p38, and ERK1/2, while inhibits STAT3, whose activation positively affects tumor cell proliferation, survival, angiogenesis and invasion [29]. Finally, Acrp30, through the suppression of NF-kB phosphorylation, suppresses the pro-inflammatory and anti-apoptotic related properties [16,30].

Despite the fact that Acrp30 mainly affects glucose homeostasis and fatty acids metabolism in liver, muscle and adipose tissue, other different cell processes can be influenced in the same way [28]. Precisely, Acrp30 suppresses pro-inflammatory cytokines production, such as TNF-α and IL-6, C reactive protein and growth factors, and induces several anti-inflammatory molecules, mainly IL-10 [31]. Together with anti-inflammatory properties, Acrp30 also inhibits monocyte adhesion, macrophage transformation and proliferation of smooth muscle cells [32].

## 3. An Adipokine with Anticancer Action

The hypothesis that adipose tissue is involved in tumorigenesis is now called “adiponcosis” [33,34]. In this regard, identification of obesity as a risk factor in malignancies certainly represented the first evidence of the adipokine’s dysregulation involvement in cancer outcome [35]. The obese condition is often associated with an increase of infiltrating immune cells which, invading the adipose tissue, provoke unbalance in pro-inflammatory cytokines and adipokines, thus supporting tumor development [36]. Several cytokines, such as IL-1, IL-6 and TNF-α, and different adipokines, mainly leptin and Acrp30, have revealed a pronounced ability in controlling proliferation and invasion of cancer cells [37].

Even though nowadays we know how the obesity-related cancer risk may vary depending on multiple factors, such as body site occurrence, fat distribution and adipose-derived profiling, a copious number of studies have largely investigated the Acrp30 role in cancer, even besides the metabolic-related disorders [29]. In this regard, dysregulation in Acrp30 levels has been reported to be negatively associated with breast cancer occurrence in both premenopausal and postmenopausal women [34,38]. Besides being correlated with an increased risk of developing colorectal cancer, low Acrp30 serum concentrations have also been linked to tumor-stage and poor prognosis in this specific tumor type [8,39]. Similarly, a significant inverse correlation has been observed in many other tumors, including gastric, endometrial, pancreatic and renal cell carcinoma [16].

Contrasting results already co-exist in lung cancer, instead. Speaking of which, whilst some studies indicate no significant association between Acrp30 levels and lung cancer, others strongly support the hypoadiponectinemia as a lung cancer progression clinical sign [40,41,42]. In this connection, very recently, Nigro and coworkers reported a substantial reduction of total adiponectin levels in non-small-cell lung cancer (NSCLC) patients compared to healthy subjects, markedly affecting the high molecular weight [7]. Moreover, they also observed a higher AdipoR1 expression and a lower T-cadherin level in those patients. Similarly, in thyroid cancer (TC), although several epidemiologic studies reported that both obesity and low circulating Acrp30 levels are positively associated to TC occurrence, Abooshahab et al. found no differences in Acrp30 levels between TC patients and cancer-free controls [43].

In preclinical cancer models, Acrp30 administration strongly impacts on both cell viability and death, even though, as well as for serum-related data, there are also confounding elements [33]. In breast cancer, for instance, Acrp30 induces a dichotomic effect stimulating growth in ERα+ MCF-7 cells and inhibiting proliferation of ERα− MDA-MB-231 cells. Much lighter is the status in lung cancer, where Acrp30 treatment induces growth inhibition and apoptosis through pAMPK/mTOR pathways and CREB downregulation [14,15]. Similarly, Acrp30 reduces cell proliferation rate even in colorectal cancer via AMPK and ERK1/2 activation [44,45]. More recently, our group has demonstrated that Acrp30 treatment inhibits cell proliferation and cell viability of BCPAP and K1 thyroid cancer cells in a time- and dose-dependent manner. Additionally, Acrp30 has also been found to inhibit thyroid cancer cell motility and invasion capacity [46]. Despite other related findings supporting the relevance of Acrp30 in cancer, the extent of remaining preclinical studies and the limited available types make unfeasible a complete overview.

Nevertheless, it is important to take into account that the Acrp30-related antiproliferative properties are often paired with inflammation and/or oxidative stress modulation in cancer, even though results are not always in accordance [15,47].

## 4. The First Adiponectin Receptor Agonist: From the Discovery to the Anticancer Findings

Starting from its discovery in 2013, the speculative therapeutic usage of AdipoRon has become factual even in cancer. As a result of chemical library screening at the Open Innovation Center for Drug Discovery (The University of Tokyo), AdipoRon has been identified as the first orally active adiponectin receptor agonist thanks to its ability of turning on AMPK and bonding AdipoR1 and AdipoR2 in C2C12 murine myeloblast cells [48]. Specifically, more than nine thousand compounds were initially selected based on motif similarities with both GPCR ligands and AMPK activators. 2D and 3D pharmacophore analysis was subsequently applied with the purpose of skimming off ineligible structures and redundant hitters. Finally, assessing the ability to phosphorylate AMPK in both proficient and siRNA-induced AdipoRs deficient C2C12 cells, Okada-Iwabu and colleagues recognized the compound n. 108049, namely AdipoRon, as the top score and the highly active adiponectin receptor agonist.

Synthesized by Enamine Ltd. (Kiev, Ukraine), starting with alkylation of hydroxybenzophenone with methyl chloroacetate, AdipoRon consists of three distinct functional groups arranged together as follows: 1-benzyl 4-substituted 6-membered cyclic amine moiety, carbonyl group and a terminal aromatic ring. Radioactive binding and scatchard analysis further confirmed the AdipoRon specificity in binding both AdipoR1 and AdipoR2 in vitro with a constant value for the dissociation (K_d_) of about 1.8 and 3.1 μM, respectively.

Unfortunately, the missing of AdipoRs crystal structure hampered the proper recognition of the binding site during discovery and, albeit the 3D receptors’ shape has currently been defined, this information remains largely unknown. Certainly, the suitable distance between the carbonyl group and the cyclic amine, as well as within the cyclic amine and the aromatic ring(s), could provide the right spatial arrangement for both AMPK activation and AdipoR-dependency. Moreover, since specific structural motifs have also been observed in other GPCR ligands, such as the aromatic rings binding via cyclic amine, similarities in ligand recognition cannot be ruled out between these two types of membrane receptors.

AdipoRon has been proven to possess pharmacological properties similar to Acrp30, exhibiting strong anti-obesity, anti-diabetic, anti-depressant, anti-ischemic and anti-hypertrophic features over the years [20]. Moreover, it also improves pathological conditions like post-traumatic stress disorder, anxiety and systemic sclerosis [49,50,51].

More recently, several studies have conferred to AdipoRon marked anticancer properties in different preclinical cancer models, particularly in pancreatic ductal adenocarcinoma (PDAC), myeloma, breast and ovarian cancer [52,53,54,55].

In addition, despite the fact that no study has directly investigated the effect of AdipoRon in colorectal cancer (CRC), Malih and Najafi proposed a hypothetical model in which Acrp30 and AdipoRon had a similar behavior in suppressing CRC cell growth, hinting at the employment of this synthetic compound in obesity-related CRC chemoprevention [56]. To corroborate this assumption, organoids from low-fat mice treated with AdipoRon exhibited a reduction in Lgr5+ cells, a well-known marker of long-lived cycling stem cells capable of driving intestinal cancer [57]. Interestingly, performing an analogue stimulation in high-fat mice implicated a reduction in AdipoRon efficacy, supposing a potential diet-induced responsiveness. Very recently, Takenaga et al. obtained significant results along these lines. Indeed, the concomitant presence of obesity-associated factors weakened the AdipoRon-mediated anticancer effects in PDAC, since both body weight and orthotopic tumor growth were slightly affected in diet-induced obese prediabetic mice by that kind of Acrp30 agonist [58].

Based on the existing knowledge, only some AdipoRon-related mechanistic aspects become oblivious, while many others remain unknown. Speaking of which, whilst a G0/G1 phase delay or block seems to be the functional key mechanism by which AdipoRon induces growth arrest in cancer models, the cytotoxic-mediated effects are not entirely clear [53,59]. Indeed, although an increase in apoptosis-related proteins has been observed in reaction to AdipoRon administration in PDAC, myeloma and ovarian cancer, programmed cell death does not appear to be the only signaling engaged in AdipoRon-mediated toxicity and, moreover, contrasting evidence also exists regarding the apoptosis involvement, especially in PDAC [52,53,54]. In this respect, characterizing AdipoRon consequences in different human and mouse pancreatic cancer models, Messaggio and colleagues recognized the Annexin V positive cell increase as a direct marker of apoptosis induction [52]. On the contrary, using Z-VAD-FMK as a pan-caspase inhibitor, Akimoto and coworkers did not observe any amelioration in AdipoRon-induced cytotoxicity, thus excluding caspase-dependent apoptosis as the leading cause of death [59]. Beyond apoptosis, other different types of cell death have been described after AdipoRon administration in cancer models, however, including RIPK1/ERK-dependent necroptosis and AMPK-mediated autophagy [54,59].

Mimicking Acrp30, AdipoRon activates AMPK and its related downstream target Acetyl-CoA carboxylase (ACC), whereas AMPK-dependent mechanistic target of rapamycin (mTOR) inhibition has only been excluded in ovarian cancer cells [53]. Nevertheless, beyond the canonical Acrp30-related signaling, several AMPK-dependent and independent pathways have also been reported to be impacted by AdipoRon. In PDAC, for instance, a long-term signal transducer and activator of transcription 3 (STAT3) inhibition and a very early extracellular signal-regulated kinase 1/2 (ERK1/2) activation have been observed [52,59].

## 5. AdipoRon Exerts Antineoplastic Properties in Osteosarcoma Models

The consolidated available findings recognize both Acrp30 and AdipoRon as potential anti-tumoral agents in a wide variety of cancer types, even affecting organs with a different embryonic origin layer. Surprisingly, there were no studies in osteosarcoma, the most frequently occurring bone malignancies in young people [60]. Therefore, in the wake of the promising AdipoRon results, we recently decided to investigate its possible consequences in this bone-related tumor. In order to support an understanding and correct reading of the data, in this section we briefly summarized the main findings and tests performed in our latest publication [61].

In depth, using U2OS, MG-63 and Saos-2 as representative human osteosarcoma models, we firstly assessed their relative protein expression in Acrp30 receptors AdipoR1, AadipoR2 and Cadherin-13. Consistent with existing evidence, osteosarcoma cells expressed detectable levels of adiponectin receptors, and thus they were proficient in responding to AdipoRon stimulation.

**Figure 1:**
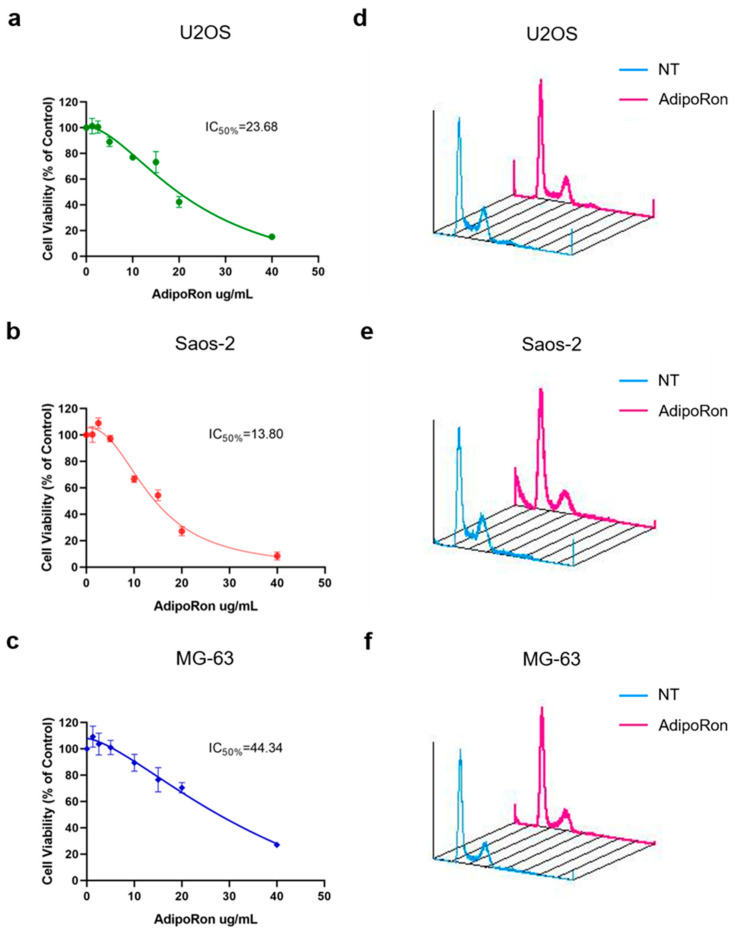
Effects of AdipoRon treatment on cell viability and cell cycle distribution in Osteosarcoma cells. U2OS (**a**), Saos-2 (**b**) and MG-63 (**c**) were supplemented with AdipoRon (from 1.25 μg/mL to 40 μg/mL) for 72 h in order to assess both cell viability and IC50 by MTT assay. U2OS (**d**), Saos-2 (**e**) and MG-63 (**f**) were grown in presence and absence of 20 μg/mL AdipoRon for 48 h and then compared for cell phase distribution by FACS analysis. Data reported in the figure above have already been published [61].

Successively, in order to test its pharmacological usage in osteosarcoma, we treated U2OS, MG-63 and Saos-2 cells with increasing AdipoRon concentrations for 72 h and then we performed MTT assays with the purpose of estimating both cell viability and half-maximal inhibitory concentration (IC50). In accordance with previous studies in which AdipoRon is proposed as antiproliferative compound, we covered a wide concentration spectrum, ranging from 1.25 to 40 µg/mL. Starting from 10 µg/mL, AdipoRon caused a significant cell growth inhibition in all tested osteosarcoma cells, even though, as revealed by IC50 quantification, a different sensitivity has clearly been observed among them (Figure 1a–c). Specifically, Saos-2 (IC50 = 13.80 µg/mL) and MG-63 (IC50 = 44.34 µg/mL) were defined as the most and the less reactive, respectively.

With the purpose of connecting AdipoRon-mediated anticancer properties to specific growth impairment, we compared treated and untreated cells for nuclear DNA content using flow cytometry analysis. In agreement with previous findings, propidium iodide staining of AdipoRon-supplemented cells revealed both G0/G1 phase intensification and S phase drop in all three osteosarcoma cell models (Figure 1d–f).

The different responsiveness unveiled by MTT assay among U2OS, MG-63 and Saos-2 was further confirmed by cell phases’ assessment. Speaking of which, besides the different magnitude in slowing growth, AdipoRon administration triggered sub-G1 appearance only in Saos-2, suggesting a potential cytotoxic effect in this osteosarcoma model. Whilst the onset of cleaved-caspase 3 (CC-3) and microtubule-associated protein 1A/1B-light chain 3 (LC-3) justified sub-G1 occurrence, they did not delineate the type of cell death involved in AdipoRon-mediated cytotoxic features. Based on both our and existing results, we cannot exclude a multiple contribution of different forms of cell death in reaction to AdipoRon in Saos-2, including apoptosis and autophagy.

From a mechanistic point of view, we detected a sudden ERK1/2 phosphorylation in Saos-2 and in U2OS already after 15 min of AdipoRon exposure. Concurrently, ERK1/2 activation led to p70S6K downregulation in Saos-2 but not in U2OS cells. Taking into account the different sensitivity between these two models, we speculated the involvement of p70S6K, a piece of mTORC1 signaling pathway, in AdipoRon-mediated cell death. To achieve this, we treated U2OS with mTORC1 selective inhibitor Everolimus alone and in combination with AdipoRon. Unfortunately, despite a massive G0/G1 accumulation, co-treatment did not provoke sub-G1 manifestation in U2OS. Moreover, no additive effects in slowing growth, as well as in sub-G1 accumulation, were observed in response to Everolimus plus AdipoRon in Saos-2.

**Figure 2:**
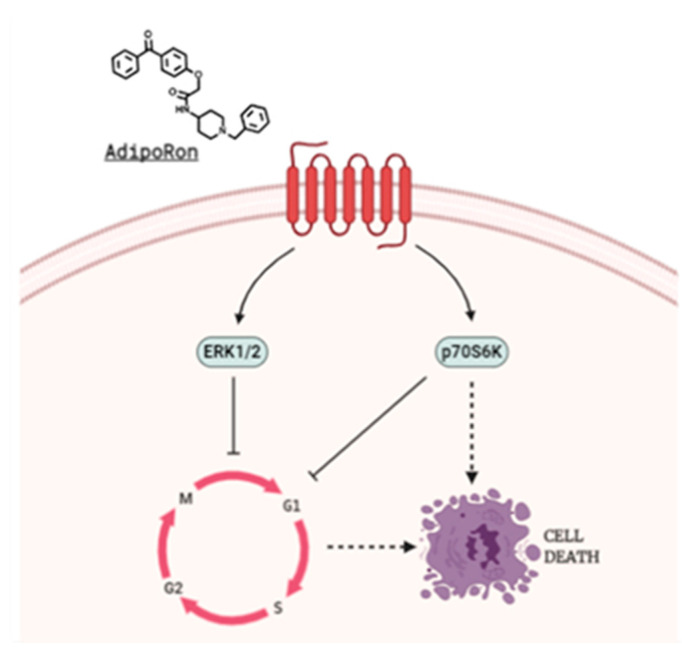
Theoretical proposed AdipoRon-mediated mechanism of action in Saos-2 Osteosarcoma cells.

Collectively, our findings firstly proposed AdipoRon as an anticancer compound in osteosarcoma, even though, as for much other related evidence, the underlying molecular mechanisms remain murky. In this regard, despite the fact that ERK recognizes a well-known oncogenic and mitogen pathway, the duration of its activation, magnitude and compartmentalization can implicate even opposite effects, including cell cycle blockage and apoptosis induction [62,63]. Strangely, mTOR and mTORC1 modulation, which could represent the “natural” AdipoRon-related downstream, would not seem to be implicated in U2OS. Considering its involvement in both apoptosis and autophagy, p70S6K could mediate AdipoRon-induced cell death in Saos-2, instead (Figure 2) [64,65]. Nevertheless, more comprehensive studies are required and we are currently working on this.

## 6. The Interplay between Metabolism and Cancer: Unexplored Targets and Future Challenges within AdipoRon

AdipoRon-related metabolic adaptive responses have marginally been addressed in cancer models, even though, considering the Acrp30 relevance in regulating energy homeostasis and, more generally, cell metabolism, they are likely to occur as cause or effect of the anticancer-mediated consequences. In this respect, Akimoto and coworkers firstly described an AdipoRon-induced mitochondrial dysfunction in MIAPaca-2 cells, identifying Ca^2+^ overload and superoxide production as potential reasons of this failure [59]. More recently, Salinas and coworkers have brought to light an AdipoRon-mediated change in cell membrane rigidity due to a reduction in cholesterol content, which results in free cholesterol accumulation in lysosomes [66].

Modulation in mitochondrial activity and ATP production has also been detected in non-tumor cells when exposed to AdipoRon, however [67]. Interestingly, Grandhaye and coworkers reported in human luteinized granulosa cells a significant AdipoRon-mediated drop in aromatase expression and estrogens secretion. Due to its key role in estrogens’ biosynthesis, aromatase has been recognized as a crucial target in hormone-dependent cancers [68]. Despite being highly effective, aromatase inhibitors generally lead to cardiovascular events increase and bone density reduction [69]. Moreover, selective estrogen receptors modulators, which mainly represent the alternative therapeutic approach in these tumors, have shown additional issues in long-term therapies, such as increased risk of endometrial cancer and drug resistance [70]. Therefore, considerable efforts have been made in identifying novel effective compounds with less side effects over the years. In light of these findings, AdipoRon could be explored as a potential aromatase regulator in estrogen-responding tumors with the purpose of implementing the range of therapeutic options.

As widely documented in several non-tumor studies, AdipoRon-mediated AMPK phosphorylation usually precedes the peroxisome proliferator-activated receptor gamma coactivator 1 alpha (PGC-1α) activation [71,72,73]. Considered as a master regulator of energy metabolism, PGC-1α controls both mitochondrial biogenesis and oxidative phosphorylation [74]. Even though stress and environmental conditions influence PGC-1α expression, its clinical significance remains quite disorienting in cancer [75]. In this connection, dichotomous effects have been reported in malignant disorders and, occasionally, even within the same tumor subtypes, leaving many open questions about its contribution in tumor initiation and maintenance [76]. Considering its ability in modulating both PGC-1α and cancer cell growth, AdipoRon could be employed as a useful tool to better define the PGC-1α role tumor by tumor.

Investigating the AdipoRon role in preventing type 2 diabetic nephropathy in db/db mice, Choi and colleagues observed a robust downregulation in both protein phosphatase 2A (PP2A) and ceramide levels, which resulted in ceramide-to-sphingosine-1 phosphate ratio normalization [77]. In the cancer background, ceramides and PP2A could constitute an intriguing duo to better comprise the AdipoRon-mediated anticancer properties. In this respect, both ceramides and PP2A have been shown to act as potent tumor suppressors, triggering apoptosis, autophagy and cell cycle arrest [78]. As a ceramide downstream pathway, indeed, PP2A phosphorylation (activation) promotes both p27 accumulation in cell cycle arrest and GSK-3β mediated apoptosis induction [79].

## 7. Beyond AdipoRon: A Journey through Novel and Existing Compounds

Aside from AdipoRon, other promising Adiponectin Receptor Agonists have been discovered over the years. By the time AdipoRon took shape, an Acrp30 analog, capable of mimicking its active site, was developed and named ADP355 [80]. This peptidomimetic compound has been demonstrated to inhibit cell growth in chronic myeloid leukemia, breast and prostate cancer [80,81,82]. Rather than AdipoR2, ADP355 modulates several intracellular pathways through AdipoR1 binding, including AMPK, mTOR, ERK1/2 and STAT3. Considering the multimeric Acrp30 structure, and its related high biological activity, in a follow-up study, Otvos and collaborators developed a linear branched ADP355 dimer, namely ADP399 [82]. Compared to the monomeric ADP355, ADP399 was 20-fold more effective in inhibiting K562 CML and MCF-7 cell growth.

Among the plant-related products, two distinct compounds have been proven to possess structural and functional homologies with respect to Acrp30, specifically GTDF and Osmotin [83,84]. 6-C-β-d-glucopyranosyl-(2S,3S)-(+)-5,7,3′,4′-tetrahydroxydihydroflavonol, simply known as GTDF, was firstly recognized as an analog of the dietary flavonoid quercetin, even though, in a competition radioligand assay performed in C2C12 myotubes, it showed ability of interacting with AdipoRs [83]. Different studies identified GTDF as an alleviating factor against skeletal muscle atrophy and osteoporotic bone, whereas no cancer studies have been reported yet [85,86]. Similarly, whilst Osmotin seems to be effective in counteracting obesity, diabetes and cardiovascular diseases, there is no anticancer proof described so far [73].

Lately, based on gAd and gAd-derived peptides strategy, Xu and colleagues designed and synthetized a series of AdipoR1/AdipoR2 dual agonists, indicating JT003 as the most powerful one [87]. Indeed, comparing JT003 with other AdipoRs agonists, they observed strong-related anti-lipogenesis and anti-fibrogenesis properties, and a better half-life duration. Similarly, very recent studies have focused on identifying more effective and selective agonists, using AdipoRon as the opening template [88,89]. In this connection, Qiu and coworkers recognized a novel Acrp30 receptor agonist with remarkable anti-inflammatory properties, as documented in LPS-induced endotoxemia and diet-induced obesity mouse models. As a final outcome of nuclear factor kappa B (NF-κB), mitogen-activated protein kinase (MAPK) and c-Maf inhibition, phlogistic processes were mainly relieved through limited pro-inflammatory cytokines production [88]. To all of these new compounds, no tests have been carried out in preclinical cancer models. Table 1 reports the most relevant findings obtained by using adiponectin receptor agonists in cancer models.

## 8. Conclusions

Despite the fact that copious findings confer to Acrp30 the title of “anti”, referring to its ability of mitigating different pathological conditions, such as inflammation, diabetes, arteriosclerosis and cancer, no Acrp30-based therapies are currently available in clinical practice. Difficulties in converting the full-size Acrp30 into a viable drug formerly represented the main limitation for its medical landing. In this regard, discovering small and synthetic agonists has provided new potential therapeutic options in treating those sicknesses.

Although initial preclinical results underline the agonists’ capability of exerting antiproliferative effects in several cancer models, many issues concerning the related molecular mechanisms remain unknown. Likewise, albeit Akimoto and colleagues displayed a slight AdipoRon ability in reducing both normal epithelial HPAEpiC cell viability and body weight in mice, very few data are currently available regarding the “safety” of this class of compounds in non-tumor tissues [59].

However, potentially valuable results in “degree-tolerance”, at least as far as adiponectin agonist peptidomimetics, are expected very soon. Speaking of which, in 2019, Allysta Pharmaceuticals initiated a Phase 1/2a clinical trial aimed to evaluate the safety and to explore the efficacy of two distinct concentrations of ALY688 agonist, also referred to as ADP355, in dry-eye disease (NCT04201574) and reporting data are expected to be revealed no later than 31 October 2021.

Conversely, the issues related to the small molecule of adiponectin receptor agonists could be more intricate. Indeed, small molecules are typically marked by unintended, often unknown, biological targets which provoke both preclinical and clinical toxicity [90]. In this connection, whilst cancer therapy is becoming increasingly focused on multi-target therapy, there is also the increased possibility of side-effects as a consequence of promiscuous binding properties [91]. Despite the fact that no evidence currently describes aspecific bindings between AdipoRon and other receptors besides adiponectin, considering both its chemical structure and the persistent failure in recognizing the exact binding site, this chance cannot be ruled out. The employment of structural biology and computational methods can be useful tools to predict the off-targets effect, as well as the phenotypic outcome, of these compounds however.

Surely, the identification of highly selective and specific compounds could further push the adiponectin receptor agonists’ approval in clinical. In this connection, the existence of three distinct Acrp30 receptors and two main downstream targets, each consisting of several isoforms with a different tissue distribution and function, could represent a breeding ground to design novel/derivate molecules and to restrict their working spectrum, respectively. To corroborate this assumption, it is interesting to note how the latest findings are focusing in precisely this direction [88,89].

In conclusion, the promising results obtained so far make this class of medication an intriguing feasible strategy to treat cancer, even though it is appropriate to clarify that their clinical employment does not appear so close in the next future. Expressing our personal view regarding the strengths and weaknesses of adiponectin receptor agonists, many shortcomings come to light from the available studies and, therefore, further studies are absolutely needed to better characterize adiponectin receptor agonists, especially in the living system.

## Figures and Tables

**Table 1:** Adiponectin receptor agonists related findings in cancer models.

Agonist	Cancer Type	Cell Line	Anticancer Action	Pathway	Reference
**AdipoRon**	**PDAC**	Panc-1, Mia Paca-2 (human) Panc.02, P-4313, K-8484 (mouse)	Apoptosis	STAT3 ACC	[53]
		AsPC-1, Mia Paca-2, BxPC-3, Panc-1 (human)	G0/G1 Blockage Necroptosis	AKT ERK1/2 p38 AMPK	[60]
	**Ovarian**	OVCAR3, OVCAR4, A2780 (human)	G0/G1 Blockage Apoptosis	AMPK	[54]
	**Myeloma**	Sp2/0-Ag14 and MPC-11 (mouse)	Apoptosis Autophagy	AMPK ACC	[55]
	**TNBC**	MDA-MB-468, MDA-MB-231, LM2 (human)	n/a	n/a	[56]
	**Osteosarcoma**	U2OS, MG-63, Saos-2 (human)	G0/G1 Blockage	ERK1/2	[62]
**ADP355**	**Prostate**	LNCaP (human)	n/a	AMPK mTOR p53	[82]
	**TNBC**	MCF7, MDA-MB-231 (human)	n/a	AMPK ERK1/2 STAT3	[81,83]
	**Myeloid**	K562 (human)	n/a	n/a	[83]
	**Glioma**	LM18 (human)	n/a	STAT3 AKT	[81]

(n/a) Not Available.
